# Robotically assisted hybrid coronary revascularization—Masterly technique but is it for the masses?

**DOI:** 10.1111/jocs.16241

**Published:** 2022-01-20

**Authors:** Pradeep Narayan, Gianni D. Angelini

**Affiliations:** ^1^ Rabindranath Tagore International Institute of Cardiac Sciences, Narayana Health Kolkata West Bengal India; ^2^ Bristol Heart Institute Bristol University Bristol UK

**Keywords:** hybrid coronary revascularization, OPCAB, robotically assisted CABG

## Abstract

Hybrid coronary revascularization consists of left internal thoracic artery (LITA) graft to the left anterior descending (LAD) artery and transcatheter revascularization of the non‐LAD stenosis in specific settings to achieve complete coronary revascularization. Technique to perform the LITA to LAD graft has ranged from median sternotomy with cardiopulmonary bypass to robotically assisted totally endoscopic coronary bypass surgery using beating heart revascularization.

Hybrid coronary revascularization (HCR) first described in 1996^1^ consists of left internal thoracic artery (LITA) graft to the left anterior descending (LAD) artery and percutaneous coronary intervention (PCI) of the non‐LAD stenosis in specific settings to achieve complete coronary revascularization. Technique to perform the LITA to LAD graft has ranged from median sternotomy with cardiopulmonary bypass to robotically assisted totally endoscopic coronary bypass surgery using beating heart revascularization. Torregrossa et al. in this study on HCR harvested the LITA robotically and performed the LITA to LAD anastomosis through a mini‐thoracotomy without using cardiopulmonary bypass.^2^ They have also compared this technique of HCR with conventional on‐pump coronary artery bypass grafting (CABG) and off‐pump coronary artery bypass (OPCAB). The authors have shown excellent outcomes with all three techniques. In this study, HCR led to reduced postoperative bleeding, need for blood transfusion and re‐exploration for bleeding as well as reduced incidence of atrial fibrillation (AF). This resulted in shorter lengths of intensive care unit (ICU) and hospital stays. The long‐term survival was similar to conventional on‐pump CABG and OPCAB. To balance the baseline differences that existed between the groups, the authors used inverse probability of treatment weighting, which is another strength of the paper and makes the conclusions drawn more reliable.

The reduction in postoperative blood loss along with reduced blood transfusion requirements is one of the most consistent advantages reported and has been confirmed in several meta‐analyses.^3^, ^4^, ^5^, ^6^, ^7^ Equally, most of the studies have also confirmed that compared with other techniques of revascularization, HCR is associated with similar 30‐day mortality.^3^, ^4^, ^5^, ^6^, ^7^ The evidence on reduction in rates of AF is not very strong with other studies and meta‐analyses reporting no difference in AF rates between HCR and other techniques of revascularization.^5^, ^8^ The shorter ICU and overall hospital length of stay reported in this study are also supported by several other studies.^4^, ^5^, ^6^, ^7^ However, the definition of what constitutes operative time and hospital length of stay needs further discussion. HCR consists of two distinct procedures—CABG and PCI. In a large number of patients, HCR is carried out as a staged procedure during two separate admissions and the hospital length of stay should include a combined duration of stay for both the interventions. Similarly, the operative time of HCR, unless carried out as a single‐stage procedure, should include the time for both the surgical and transcatheter components. Regardless, most of the studies have shown that the operating time with HCR is significantly longer irrespective of whether they are robotically assisted or not.^8^, ^9^ HCR constitutes a very wide range of approaches and the methodology of studies on HCR, needs to provide more granularity. Whether the HCR was a single or two‐staged procedure, whether PCI preceded CABG or vice versa, the duration between the two procedures, any complications during waiting for the second intervention, and whether it was planned or unplanned must all be reported to make a more comprehensive and accurate comparison. The importance of this granularity cannot be overemphasized as, in the absence of data regarding both the components of HCR, it is difficult to draw reliable inferences both from scientific as well as resource utilization perspectives.

Robotically enhanced HCR is a niche area of coronary revascularization that requires the highest degree of technical expertise. Unlike PCI alone, it accomplishes revascularization of the LAD with the best graft, the LITA, and thus provides the best of both worlds. The technique needs to be evaluated from a clinical outcome perspective but also from the economic perspective as well as the patient's perspective. From an economic perspective, studies attempting the cost–benefit assessment have shown considerable variations based on the surgical access or utilization of robotic assistance. Two studies with a similar design, where HCR was carried out as a single‐stage procedure using mini‐thoracotomy for a LITA to LAD anastomosis, reported a nonsignificant increase in costs associated with HCR when compared to conventional OPCAB.^10^, ^11^ However, it has to be noted that none of these studies utilized robotically assisted LITA harvesting. Another study which compared HCR without robotically assisted LITA harvest, but used mini‐sternotomy rather than mini‐thoracotomy reported a significant increase in costs involved with HCR.^8^ Studies reporting cost‐comparison in studies similar to that of Torregrossa et al.,^2^ where LITA was harvested with robotic assistance and the anastomosis performed through a mini‐thoracotomy, have shown that irrespective of whether HCR was performed as a one‐stage single day procedure or on two separate days during the same admission the costs were found to be significantly higher.^8^, ^12^ This is not surprising at all, as both the fixed and variable costs with the robotic systems and hybrid operating rooms are bound to be higher.

From the patient's perspective, assessments are generally done based on pain scores, quality of life assessment, and markers of functional recovery like the ability to return to work. While one study showed that the pain scores after robotically assisted HCR were surprisingly similar with OPCAB^9^ the overall satisfaction scores and the odds of returning to work within the first month were significantly higher after robotically assisted HCR.^9^, ^13^ The average time for returning to complete normal activity has been reported to be shorter by roughly 3 weeks after robotically assisted HCR.^9^

Clinical outcomes constitute the most important aspect of comparison for any intervention and are evaluated by the feasibility and safety of the procedure in the short term as well as longer‐term outcomes. The safety and feasibility of HCR have now been well‐established through different studies and several meta‐analyses. Two randomized controlled studies have been published comparing HCR with CABG. The 5‐year report of the HYBRID (hybrid revascularization for multivessel coronary artery disease) trial published recently reported similar all‐cause mortality, myocardial infarction (MI), repeat revascularization, stroke, and major adverse cardiac and cerebrovascular events (MACCE) between HCR and CABG.^14^ Interestingly while the RCT confirmed clinical equipoise it also failed to show any differences in terms of reduced blood transfusion requirements or length of stay between HCR and CABG.^15^ Whether this represents a “true lack” of difference between the techniques or is an effect of “trial settings” which are often more rigid and different from real‐life selection criteria remains unclear. Another RCT, the Hybrid coronary REvascularization Versus Stenting or Surgery (HREVS), that randomized patients to HCR, CABG, and the third arm of multivessel PCI also confirmed clinical equivalence among the three techniques with respect to all‐cause mortality, rates of MI, stroke, MACCE rates and reinterventions at 3‐year follow‐up. However, once again, the trial showed no difference between HCR and CABG in terms of length of stay.^16^

Besides, both these RCTs have brought out an important issue, which is the conversion rate among patients designated to undergo HCR. In the HYBRID trial, only 93.9% of patients randomized to HCR actually received the intervention. Six (6.1%) required a sternotomy and two (2.04%) had failed PCI.^15^ Nearly, 1 in 10 (9.8%) patients randomized to the HCR arm of the HREVS trial was converted to CABG using median sternotomy.^16^ This is an important observation that further highlights the fact that while robotically assisted HCR may have good results in experienced hands it may have a long learning curve, that limits its uptake. Besides, the absence of data on conversions among previously published nonrandomized studies, suggests that patients who got converted to alternative techniques may not have been included in the analysis of these studies.

The need for specialized operative infrastructure and specialized hybrid suites has been an important deterrent for robotically assisted HCR. Robotically assisted HCR is appealing as far as patient satisfaction is concerned, however, the reported advantage of reduced requirement for blood transfusion and shorter hospital length of stay are now being challenged by RCTs. This in turn negates the economic benefit argument with HCR even further. The conversion rates associated with HCR bring the issue of expertise into the discussion as well and may further deter uptake of robotically assisted HCR. In the absence of overwhelming superiority of HCR, it is unlikely that robotically assisted HCR will be practiced by surgeons widely in the near future and it is very likely to remain confined to certain centers of excellence.

## CONFLICT OF INTERESTS

The authors declare that there are no conflict of interests.
